# Prolonged gastroparesis after corrective surgery for Wilkie's syndrome: a case report

**DOI:** 10.1186/1752-1947-2-109

**Published:** 2008-04-17

**Authors:** Muhammad I Aslam, Jonathan G Finch

**Affiliations:** 1 Northampton General Hospital, Cliftonville, Northampton NN1 5BD, UK

## Abstract

**Introduction:**

Wilkie's syndrome, a rare cause of intestinal obstruction, is related to anatomical and mechanical factors associated with the reduction of retroperitoneal fat padding. The diagnostic challenges of identifying vascular constriction between the aorta and superior mesenteric artery have been answered by advances in the field of computed tomography. Despite diagnostic confusion with intestinal dysmotility syndrome, conservative therapy with nutritional supplementation is the initial approach and duodenojejunostomy is favoured if non-surgical treatment fails.

**Case presentation:**

We present a case of a 49-year-old woman with Wilkie's syndrome with persistent symptoms of gastroparesis for 15 months following corrective surgery.

**Conclusion:**

Open and laparoscopic duodenojejunostomy have been described as the best surgical treatment options for Wilkie's syndrome, but further work needs to be done for patients with refractory symptoms of gastroparesis after these corrective surgeries.

## Introduction

Wilkie's syndrome, a rare cause of intestinal obstruction, is related to anatomical and mechanical factors associated with the reduction of retroperitoneal fat padding. The diagnostic challenges of identifying vascular constriction between the aorta and superior mesenteric artery have been answered by advances in the field of computed tomography (CT). Despite diagnostic confusion with intestinal dysmotility syndrome, conservative therapy with nutritional supplementation is the initial approach and duodenojejunostomy is favoured if non-surgical treatment fails.

## Case presentation

A 49-year-old woman was investigated for intestinal dysmotility with symptoms of gradual weight loss, postprandial epigastric bloating, a sense of repletion and vomiting over an 8-month period. Since her teenage years, she had maintained a steady weight of 48 kg, but had lost 6 kg over the last 8 months. Her past medical history was remarkable for symptoms suggestive of Raynaud's syndrome, multi-joint arthralgia and an episode of anorexia 4 years previously. She started to experience symptoms of Raynaud's syndrome nearly 5 years ago when she changed her occupation and started working in the food catering industry. There was no deterioration of the symptoms of Raynaud's syndrome associated with the weight loss. There was no history of recent trauma, surgery, prolonged immobilisation or neurological illness. Her weight loss was gradual. A gastroscopy demonstrated a large residue of fluid and undigested food in her stomach with a dilated duodenum extending approximately 10 cm distal to the pylorus. Duodenal and gastric biopsies were negative. Symptoms of presumed delayed gastric emptying failed to resolve after a trial of prokinetics and proton pump inhibitors and she was admitted with hypoalbuminaemia, hypokalaemia and continued weight loss.

**Figure 1:**
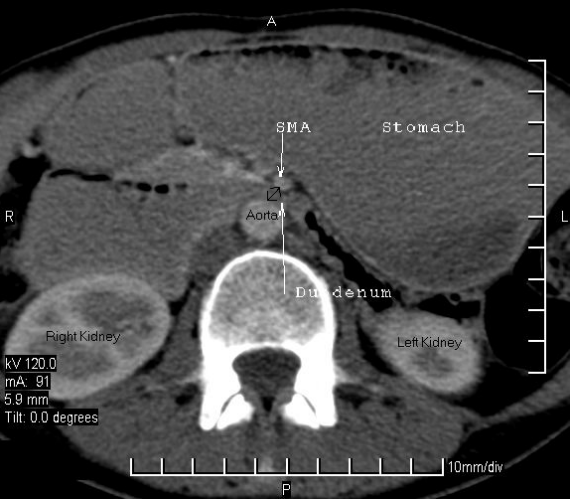
The second part of the duodenum (D2) is dilated proximal to a point of sharp obstruction at the level of the third part of the duodenum (D3).

**Figure 2:**
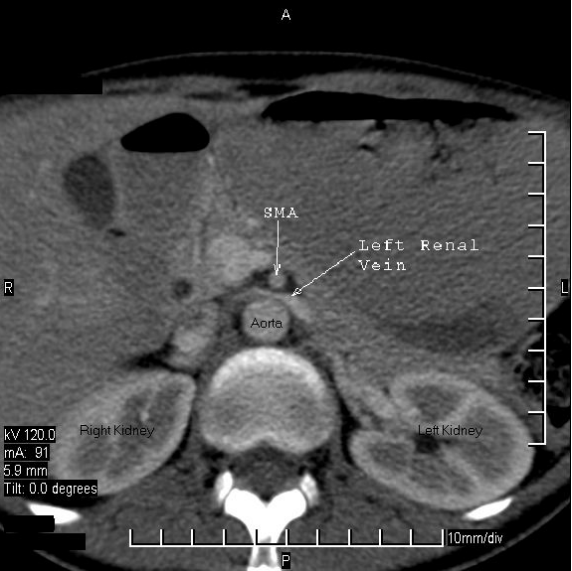
Impingement of the left renal vein between the aorta and the superior mesenteric artery.

**Figure 3:**
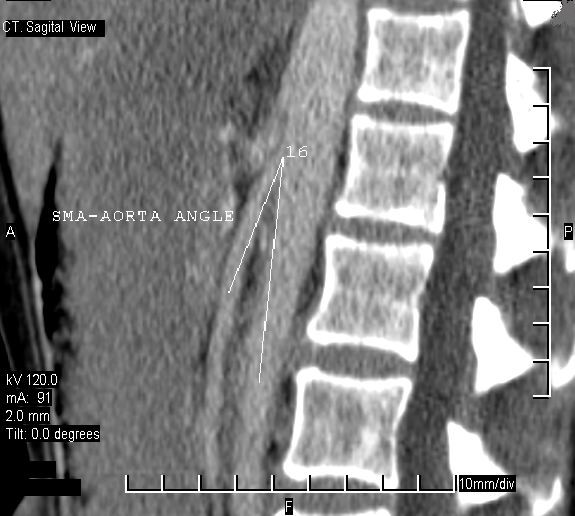
Sagittal reconstruction of CT images through the mid-abdomen showing the angle between the superior mesenteric artery and the aorta (SMA-aorta angle) is reduced to 16°.

A CT scan of the abdomen and pelvis revealed a hugely dilated stomach extending to the pelvis. The second part of the duodenum (D2) was dilated proximal to a point of sharp obstruction at the level of the third part of the duodenum (D3). At this point of obstruction, the aorto-mesenteric distance was reduced to 6 mm (Figure 1). A CT scan also confirmed the impingement of the left renal vein between the aorta and superior mesenteric artery (SMA; see Figure 2). There was no thickening of the wall of the duodenum or extrinsic mass lesion. Sagittal reconstruction of CT images through the mid-abdomen (Figure 3) showed that the angle between the SMA and the aorta (SMA-aorta angle) was reduced to 16°. A diagnosis of Wilkie's syndrome was made on the basis of clinical suspicion and CT findings. The potential cause of the syndrome was unclear and the only obvious precipitating factor was her gradual loss of weight over 8 months.

A 7-day trial of conservative management with a nasogastric tube, enteral feeding supplements and prokinetics failed. The patient's symptoms worsened, and she experienced more frequent vomiting and lost a further 1 kg. In view of the continued weight loss and worsening symptoms, the conservative management was abandoned and the patient chose to have Strong's procedure and open surgery.

An attempted laparoscopic duodenojejunostomy and mobilisation of the duodenum (Strong's procedure) was abandoned as the massively distended stomach did not allow a satisfactory assessment of the third part of the duodenum. During the open procedure (laparotomy), the duodenum was Kocherised and the duodenojejunal flexure was mobilised to perform a side-to-side duodenojejunostomy. There was no evidence of alternative causes of obstruction. Her symptoms gradually improved after the operation, she steadily gained weight and her biochemical markers returned to normal. The patient returned to work 3 weeks after surgery.

A subsequent CT scan showed less gastric distention with evidence of continued poor gastric emptying secondary to an enlarged redundant stomach, suggesting persistent gastroparesis. The symptoms of vomiting and poor gastric emptying returned 5 months after surgery. A gastroscopy confirmed an enlarged redundant stomach with a patent duodenojejunostomy and poor gastric emptying. Her symptoms improved with supplement feeding for 3 days and correction of her hypokalaemia. Endoscopic biopsies of the duodenum and stomach failed to indicate any alternative diagnosis such as intestinal myopathy.

She was also reviewed in our hospital and a tertiary hospital 14 months after the initial operation. She still had a few episodes of vomiting especially after eating a large meal and her weight remained unchanged. Again endoscopic and radiological investigation confirmed the diagnosis of gastroparesis related to Wilkie's syndrome.

## Discussion

Wilkie's syndrome is an uncommon cause of intestinal obstruction [1]. It was first described by Von Rokitansky in 1842 and popularised later by Wilkie. It is also known as SMA syndrome, cast syndrome and arteriomesenteric duodenal compression. Wilkie's syndrome is related to anatomical and mechanical factors and to acute or chronic reduction of the retroperitoneal fat. Retroperitoneal fat padding is diminished in several debilitating conditions that have marked weight loss in common.

In such situations, the angle formed by the aorta and the SMA is reduced, thus forming a vascular constriction at the point where the duodenum usually crosses, thereby triggering the syndrome [2]. It has also been reported in cases associated with spinal corrective surgery, body casts, restorative proctocolectomy and abdominal aortic aneurysm. A diagnosis of Wilkie's syndrome should be considered in adults with postprandial pain, weight loss and bilious vomiting. Historically, barium meal and arteriography were used as diagnostic tools [3] but more recently CT, CT-angiography and magnetic resonance imaging (MRI) have been used and shown higher diagnostic sensitivity. In conventional angiographic studies, the SMA-aorta angle was found to be 7° to 22° (normal: 25° to 50°) and the SMA-aorta distance was found to be 2 to 8 mm (normal: 10 to 28 mm) in this condition [4]. There is a high correlation between the reduction of the SMA-aorta distance and the severity of the symptoms [5]. The real diagnostic challenge is faced where patients present with mixed elements of anatomical compression and dysmotility.

The treatment of SMA syndrome is aimed at the precipitating factor, which is usually related to weight loss [6]. Therefore, conservative therapy with nutritional supplementation is the initial approach. Non-surgical treatment with decompression of the stomach via a nasogastric tube, supplement nutrition and feeding in a prone position is recommended as initial therapy while open duodenojejunostomy is favoured if non-surgical treatment fails [7]. Laparoscopic duodenojejunostomy is a feasible alternative option for treating Wilkie's syndrome. It provides the benefits of a minimally invasive, definitive surgical technique for duodenal obstruction [8]. Gastroparesis after correction surgery is a frequently encountered problem related to gastric and duodenal atony. Although the presence of such persistent symptoms has been described in the literature, there is little information on their management. Prokinetics have been tried with some success in patients with refractory symptoms after surgery. The other available treatment options for gastroparesis include a gastric pacemaker and gastric volume reduction surgery, but there is a lack of real evidence for their role in treating gastroparesis after corrective surgery for Wilkie's syndrome.

## Conclusion

Open and laparoscopic duodenojejunostomy have been described as the best surgical treatment options for Wilkie's syndrome, but further attention is needed to the management of patients with refractory symptoms of gastroparesis after corrective surgery. This case report emphasises the historic management of Wilkie's syndrome and opens a new debate about the management of prolonged gastroparesis after conventional duodenojejunostomy for Wilkie's syndrome.

## Competing interests

The authors declare that they have no competing interests.

## Authors' contributions

MIA designed the study, acquired the data, analysed and interpreted the reports and drafted the manuscript. JGF made substantial contributions to the conception, design, critical analysis and intellectual review of the manuscript. All authors have read and approved the final manuscript.

## Consent

Written informed consent was obtained from the patient for publication of this case report and accompanying images. A copy of the written consent is available for review by the Editor-in-Chief of this journal.
